# Machine Learning and Metabolomics to Characterize Warburg-Like Metabolic Subtypes in Human Retinal Endothelial Cells Exposed to Risk Factors Associated With Proliferative Diabetic Retinopathy

**DOI:** 10.1167/tvst.15.8.26

**Published:** 2026-08-26

**Authors:** Oase Sbei, Shaimaa Eltanani, Sara Ibrahim, Thangal Yumnamcha, Sangly P. Srinivas, Ahmed S. Ibrahim

**Affiliations:** 1 Department of Ophthalmology, Visual, and Anatomical Sciences, School of Medicine, Wayne State University, Detroit, MI, USA; 2 College of Literature, Science, and the Arts, University of Michigan, Ann Arbor, MI, USA; 3 School of Optometry, Indiana University, Bloomington, IN, USA; 4 Department of Pharmacology, School of Medicine, Wayne State University, Detroit, MI, USA; 5 Molecular Therapeutics Research Program, Karmanos Cancer Institute, School of Medicine, Wayne State University, Detroit, MI, USA

**Keywords:** proliferative diabetic retinopathy (PDR), the Warburg effect, human retinal endothelial cells (HRECs), high glucose (HG), hypoxia (Hyp), machine learning, AdaBoost, angiogenesis

## Abstract

**Purpose:**

High glucose (HG), hypoxia (Hyp), and their combination are major risk factors for proliferative diabetic retinopathy (PDR). Although these conditions induce features of the Warburg-like metabolic reprogramming in human retinal endothelial cells (HRECs), it remains unclear whether they produce distinct metabolic and angiogenic subtypes. This study aimed to characterize the Warburg-like–associated metabolic heterogeneity induced by these PDR-related risk factors and evaluate the ability of supervised machine-learning models to distinguish these subtypes.

**Methods:**

HRECs were cultured under normoglycemic, HG, Hyp (2% O_2_), and combined HG-Hyp conditions. Untargeted LC-MS/MS metabolomics quantified metabolites spanning carbohydrates, amino acids, nucleotides, and lipids. Principal component analysis (PCA) assessed overall metabolic variation, and Kyoto Encyclopedia of Genes and Genomes (KEGG) pathway enrichment analysis identified metabolic pathways associated with angiogenesis. In vitro angiogenesis assays measured endothelial tube formation and branching. Nine supervised classifiers (decision tree, logistic regression, naïve Bayes, random forest, K-Nearest Neighbors, neural network, gradient boosting, AdaBoost, and Support Vector Machine) were trained on the highest-ranked metabolites selected by the Information Gain Ratio feature-ranking approach. Model performance was evaluated using 10-fold cross-validation, leave-one-out cross-validation (LOOCV), permutation testing, and a classifier stability analysis under biologically meaningful distributional shift using an independent chemically induced hypoxia model (CoCl_2_).

**Results:**

PCA revealed partial separation of metabolic profiles across conditions, indicating different Warburg-like metabolic subtypes. The combined HG-Hyp condition exhibited enhanced angiogenic potential relative to either HG or Hyp alone. KEGG pathway enrichment analysis identified fatty acid biosynthesis and elongation among the most significantly enriched pathways in HRECs under combined HG-Hyp conditions, alongside amino sugar and nucleotide sugar metabolism, glycerophospholipid metabolism, the pentose phosphate pathway, and glycolysis/gluconeogenesis. Supervised machine-learning classifiers distinguished these metabolic subtypes, with AdaBoost and gradient Boosting showing the most balanced, reproducible performance across 10-fold cross-validation, LOOCV, and permutation testing, and remaining the most reliable classifiers under domain-shift testing (area under the curve = 0.88, *P* = 0.0061).

**Conclusions:**

In this exploratory analysis, HG, Hyp, and their combination drive metabolically and functionally distinct subtypes of Warburg-like metabolic reprogramming in HRECs, with HG-Hyp in combination producing a highly angiogenic phenotype. Boosting-based ensemble classifiers provide a promising framework for detecting these subtypes even under domain-shift conditions, warranting validation in larger independent datasets.

**Translational Relevance:**

Integrating metabolomics with machine-learning classification offers a strategy to identify Warburg-like metabolic subtypes in retinal endothelial cells, providing insights into angiogenic mechanisms and guiding the development of targeted diagnostics or therapeutics for PDR.

## Introduction

Diabetes is a significant public health challenge in the United States, affecting millions of individuals and leading to severe complications that reduce quality of life.^1^ Among these complications, diabetic retinopathy (DR) is a leading cause of vision loss, progressing to proliferative diabetic retinopathy (PDR) in advanced stages. PDR is characterized by pathological angiogenesis, the abnormal growth of fragile and leaky blood vessels in the retina, which can ultimately result in blindness.^2^ Despite advances in treatment, PDR remains a major cause of irreversible blindness among working-age adults, highlighting the need for improved therapeutic strategies.^3^

Current treatments for PDR, such as laser photocoagulation and intravitreal injections of anti-vascular endothelial growth factor (anti-VEGF) agents, have limitations and side effects. Laser therapy can lead to permanent peripheral vision loss and impaired night vision,^4^ whereas anti-VEGF therapies often require repeated injections, increasing the risk of complications such as retinal detachment, fibrosis, and endophthalmitis.^5^ Furthermore, these treatments do not address the underlying metabolic and molecular drivers of PDR, underscoring the need for a deeper understanding of its pathogenesis.^6^

A long-standing question in the field is why not all patients with diabetes develop PDR, even after living with diabetes for a decade.^7^ This raises the possibility that factors beyond chronic hyperglycemia play a role in driving pathological angiogenesis in PDR. Among these factors, hypoxia (Hyp) has emerged as a critical contributor, driven by reduced oxygen supply to retinal tissues due to the presence of non-perfused areas.^8^^,^^9^ Clinical studies have linked non-perfused areas to visual impairment, supported by histological and OCT evidence of neuronal cell loss,^10^ as well as fluorescein angiography (FA) evidence of neovascularization, in ischemic retinas affected by diabetes.^11^ However, the precise mechanisms by which Hyp exacerbates these diabetes-induced pathological retinal changes remain poorly understood.

Interestingly, both high glucose (HG) and Hyp are known to promote glycolytic reprogramming, albeit through distinct mechanisms.^12^ Hyp classically induces a Pasteur-like response, characterized by increased glycolysis due to limited oxygen availability. In contrast, HG can drive a Crabtree-like effect, in which elevated glucose suppresses oxidative phosphorylation and enhances glycolytic flux even in the presence of oxygen.^13^^,^^14^ Sustained exposure to these conditions can further activate transcriptional and metabolic adaptations, including hypoxia-inducible factor (HIF)-dependent pathways, leading to a persistent hyperglycolytic phenotype.^15^ This state shares key features with the Warburg effect, a metabolic phenomenon first described by Nobel laureate Otto Warburg, in which cells preferentially rely on glycolysis even when adequate oxygen is available (aerobic glycolysis) to meet biosynthetic and energetic demands.^12^

In complex disease-relevant environments such as PDR, however, the Pasteur-like and Crabtree-like programs induced by Hyp and HG are unlikely to occur in isolation. When both stressors are present simultaneously, these programs may converge into an emergent metabolic state characterized by sustained glycolytic flux coupled with enhanced biosynthetic pathway activity that supports pathological angiogenesis beyond the effect of either stressor alone. Glycolysis provides intermediates for the synthesis of proteins, nucleotides, and lipids required for cell proliferation.^16^ Throughout this study, the term “Warburg-like” is used to describe this convergent, stress-driven hyperglycolytic state, rather than implying strict constitutive aerobic glycolysis in the classical sense. Consistent with this framework, we recently demonstrated that the Warburg-like metabolic reprogramming in HRECs is not uniform; rather, HG and hypoxia each produce distinct metabolic subtypes with characteristic profiles^17^ and functional behavior.^18^ Notably, their combination gives rise to a particularly angiogenic phenotype, highlighting a non-additive interaction between these stressors.^18^ Characterizing these subtypes and the emergent state arising from their convergence may provide critical insights into PDR pathogenesis and enable more precise therapeutic targeting.

To achieve this, we conducted metabolomic analysis using high-sensitivity liquid chromatography-tandem mass spectrometry (LC-MS/MS) followed by a machine-learning approach to identify patterns within complex datasets. Nine machine learning models were used: decision tree, logistic regression, naïve Bayes, random forest, K-nearest neighbors (KNN), neural network, gradient boosting, AdaBoost, and Support Vector Machine (SVM). These models were selected to cover a range of approaches, from interpretable models to advanced methods that enhance predictive accuracy. Decision tree^19^ and logistic regression^20^ are interpretable, helping in identifying features driving classification; naïve Bayes offers computational efficiency^21^; Random Forest^22^ and gradient boosting^23^ improve predictive accuracy through ensemble tree-based learning; AdaBoost iteratively combines weak classifiers into a strong learner^24^; KNN classifies based on proximity in feature space^25^; neural network captures complex, as well as non-linear relationships among input features^26^; and SVM excels in high-dimensional data.^27^ By integrating metabolomic profiling with machine learning, this study aimed to determine the optimal classifier for distinguishing subtypes of the Warburg-like metabolic reprogramming associated with a highly angiogenic phenotype in human retinal endothelial cells (HRECs) exposed to major PDR-associated risk factors, HG and Hyp.

## Material and Methods

### HREC Culture and Experimental Conditions

Primary HRECs obtained from Cell Systems (Kirkland, WA, USA) were cultured to approximately 90% confluency in Microvascular Endothelial Cell Growth Medium-2 BulletKit (Lonza, Walkersville, MD, USA). Upon reaching confluency, the medium was replaced with 5% fetal bovine serum–containing medium lacking growth factors, and cells were treated with either 25 mM mannitol (osmotic control) or 25 mM HG for five days. The culture medium was refreshed midway through the treatment period. During the final 24 hours, cells were exposed to either normoxia or hypoxia (2% O_2_) in a humidified hypoxic chamber with 5% CO_2_. After treatment, cells were either processed for metabolomic profiling or used for in vitro angiogenesis assays.

### Angiogenesis Assay

HRECs cultured under mannitol (osmotic control), HG, hypoxia, and combined HG-Hyp conditions were assessed for angiogenic potential using a Matrigel-based tube formation assay. Growth factor-reduced Matrigel (no. 354234; Corning Inc., Corning, NY, USA) was thawed overnight at 4°C and maintained on ice before use. Fifty microliters of Matrigel was added to each well of a 96-well plate and allowed to polymerize for 30 minutes at 37°C. HRECs were detached using trypsin–ethylenediamine tetraacetic acid, resuspended in growth factor-free medium, and seeded at a density of 5000 cells per well onto the polymerized Matrigel. Cells were incubated under their respective experimental conditions for 16–18 hours at 37°C and 5% CO_2_. Tube formation was imaged using an Echo-Rebel phase-contrast microscope (ECHO Incorporated, Lake Zurich, IL, USA), and tube length and branching points were quantified using Echo-Rebel analysis tools.

### Harvesting of HRECs for LC-MS/MS Metabolomics

At the end of the treatment period, the culture medium was aspirated, and cells were gently rinsed with warm PBS to remove residual medium. Cellular metabolism was rapidly quenched by placing culture dishes in liquid nitrogen. Intracellular metabolites were extracted by adding 1 mL of pre-chilled 80% methanol to each dish, followed by cell scraping. Lysates were transferred to tubes and stored at −80°C until metabolomic analysis. Each experimental condition included eight biological replicates, yielding a total of 32 samples.

### Metabolomic Profiling

LC-MS/MS was used to profile approximately 214 metabolites spanning carbohydrate, amino acid, nucleotide, and lipid pathways in HRECs cultured under the Warburg-like-inducing conditions of HG, hypoxia, and their combination. Metabolites were extracted using a modified methanol-based protocol, dried, and reconstituted in acetonitrile-water (50:50, v/v). Profiling was performed on an AB SCIEX QTRAP 6500 LC-MS/MS system using both reverse-phase and hydrophilic interaction liquid chromatography, as previously described.^28^ Data acquisition was performed using multiple reaction monitoring in both positive and negative ionization modes, with instrument parameters optimized for broad metabolite coverage. Raw LC-MS/MS data were processed to quantify individual metabolite concentrations. For each sample, the concentration of each metabolite was normalized by dividing its concentration by the sum of the concentrations of all detected metabolites, thereby expressing each metabolite as a proportion of the total metabolite pool prior to downstream analysis. Metabolites that were not detected in any sample were excluded, leaving 165 metabolites for subsequent analysis. Feature importance was assessed using the Rank widget in Orange Data Mining software, which computed Information Gain Ratio, Gini Decrease, analysis of variance (ANOVA), and χ² scores for all detected metabolites. ANOVA returned NA for all metabolites and therefore did not contribute to feature ranking. Metabolites were ranked according to their Information Gain Ratio scores, while Gini Decrease and χ² provided complementary assessments of feature importance. To reduce dimensionality and minimize overfitting, the highest-ranked metabolites (Table 1) were retained for downstream machine-learning analysis.

**Table 1.** Feature Importance Scores of Metabolites Across Multiple Ranking Metrics

Rank	Metabolite	Gain Ratio	Gini	ANOVA	χ^2^
1	M092_deoxyinosine	0.594	0.375	NA	8.308
2	M071_N-Alpha-acetyllysine	0.586	0.375	NA	4.385
3	M105_glucosamine	0.547	0.312	NA	9.111
4	M167_Uridine 5′-monophosphate	0.547	0.312	NA	6.640
5	M307_2′-Deoxyguanosine	0.508	0.312	NA	6.429
6	M005_Kynurenic acid	0.500	0.312	NA	5.410
7	M245_Coenzyme A	0.500	0.500	NA	4.000
8	M113_Guanine	0.485	0.281	NA	3.857
9	M318_Alpha-N-Phenylacetyl-L-glutamine	0.485	0.281	NA	3.857
10	M122_Imidazoleacetic acid	0.474	0.288	NA	8.509
11	M093_Deoxyuridine	0.461	0.250	NA	5.410

### Machine Learning Analysis

Nine supervised classifiers were evaluated: decision tree, logistic regression, naïve Bayes, random forest, KNN, neural network, gradient boosting, AdaBoost, and SVM. Classifiers were trained to distinguish the four experimental conditions (control, HG, Hyp, and combined HG-Hyp) using the top-ranked metabolite features identified through feature selection (Table 1). Model performance was assessed using 10-fold cross-validation and leave-one-out cross-validation (LOOCV). For 10-fold cross-validation, the dataset was randomly partitioned into 10 approximately equal-sized folds. During each iteration, one fold was held out for testing, and the remaining nine folds were used for training. This procedure was repeated until each fold had served once as the test set, and performance metrics were averaged across all validation iterations. In LOOCV, one sample was held out for testing in each iteration, whereas the remaining 31 samples were used for training. This process was repeated until every sample had served once as the test sample, thereby maximizing data utilization and providing a near-unbiased estimate of performance for small datasets. Accordingly, performance metrics represent averages across all validation iterations rather than results from a single data split (Supplementary Fig. S1). To further ensure that observed performance exceeded random expectation, permutation testing using 100 random label shuffles was performed for selected classifiers.

Performance metrics included the area under the receiver operating characteristic curve (AUC) to assess overall discriminative ability; classification accuracy (CA) as the proportion of correctly classified samples; the F1 score as the harmonic mean of precision and recall; precision as the proportion of positive predictions that were correct; recall (sensitivity) as the proportion of true positive samples correctly identified; the Matthews correlation coefficient (MCC) as a balanced measure of classification performance that accounts for all elements of the confusion matrix; specificity as the proportion of true negative samples correctly identified; and log-loss as a measure of prediction uncertainty. Owing to the mathematical relationships among these metrics, identical numerical values may occur for some classifiers after averaging and rounding to three decimal places.

To further evaluate classifier stability under a biologically meaningful distributional shift across different hypoxia-inducing conditions, classifiers trained on physiological hypoxia (2% O_2_) were tested on a pseudo-hypoxia dataset generated using cobalt chloride (CoCl_2_, 10 µM, 48 hours) in the presence or absence of HG (four biological replicates per condition). Data handling and visualization were performed using Python with Pandas, NumPy, Matplotlib, Seaborn, and SciPy, alongside Orange Data Mining software (version 3.39) for machine learning implementation and evaluation.^29^

### Model Configuration and Hyperparameter Settings

All supervised machine-learning models were implemented with fixed hyperparameters defined a priori and held constant across all validation schemes. Deterministic (replicable) training was enabled for all stochastic algorithms to ensure reproducibility across repeated runs. Decision tree classifiers were trained using binary tree structures with pre-pruning enabled to reduce overfitting. Tree growth was constrained by requiring a minimum of three instances in both leaf nodes and internal decision nodes, with a maximum tree depth of three. Node splitting was terminated when the majority class purity reached 95%, thereby limiting further subdivision once sufficient class dominance was achieved. Logistic regression models were trained using L1 (Lasso) regularization to promote sparsity in feature coefficients and reduce model complexity in the high-dimensional metabolomic feature space. The regularization strength was set to C = 0.001, corresponding to strong penalization of large coefficients. No class weighting was applied, and model optimization assumed linear decision boundaries. Naïve Bayes classifiers were implemented under the assumption of Gaussian distributions for continuous metabolite features. Random forest classifiers were trained using an ensemble of 15 decision trees. Individual trees were restricted to a maximum depth of three to limit overfitting, and node splitting was halted when fewer than five instances remained in a node. All available features were considered at each split, and replicable training was enabled to ensure consistent ensemble construction across runs. KNN classifiers were implemented using five nearest neighbors. Euclidean distance was used as the similarity metric, and uniform weighting was applied such that all neighbors contributed equally to classification decisions. No distance-based weighting or feature scaling beyond initial normalization was applied. Neural network classifiers were trained using a feedforward architecture consisting of a single hidden layer containing 100 neurons. Rectified Linear Unit (ReLU) activation functions were used for nonlinear transformation, and model optimization was performed using the Adam optimizer. L2 regularization was applied with a regularization parameter (alpha) of 0.0001, and the maximum number of training iterations was set to 200. Replicable training was enabled to control for stochastic initialization effects. Gradient boosting models were implemented using an extreme gradient boosting (xgboost) variant. The ensemble consisted of 100 trees, each with a maximum depth of six. The learning rate was set to 0.1, and the regularization strength was set to one. The fraction of training instances used for each tree, as well as the fraction of features considered at the tree, level, and split levels, were all set to one, corresponding to full data utilization at each boosting stage. Replicable training was enforced. AdaBoost classifiers were implemented using decision trees as base estimators. The ensemble consisted of 200 boosting iterations, and the exponential loss function was used to emphasize misclassified samples during iterative reweighting. The learning rate was set to 0.5, balancing convergence speed and stability. SVM classifiers were trained using a radial basis function kernel defined as exp(−g|x – y|^2^), with the kernel parameter (g) automatically determined by the software. The regularization parameter was set to C = 1.0. Numerical tolerance for convergence was set to 0.001, and the maximum number of iterations was limited to 100.

### Data Analysis

Angiogenesis assay data were analyzed using one-way ANOVA, followed by Tukey's multiple comparisons test. Principal component analysis (PCA) was performed on detected metabolites using Python-based tools to evaluate group separation. Pathway enrichment analysis was conducted against the Kyoto Encyclopedia of Genes and Genomes (KEGG) pathway database using the Quantitative Enrichment Analysis module implemented in MetaboAnalyst 6.0.^30^

## Results

### HG, Hyp, and Their Combination Drive Heterogeneous Subtypes of the Warburg-Like Metabolic Reprogramming in HRECs

**Figure 1.**
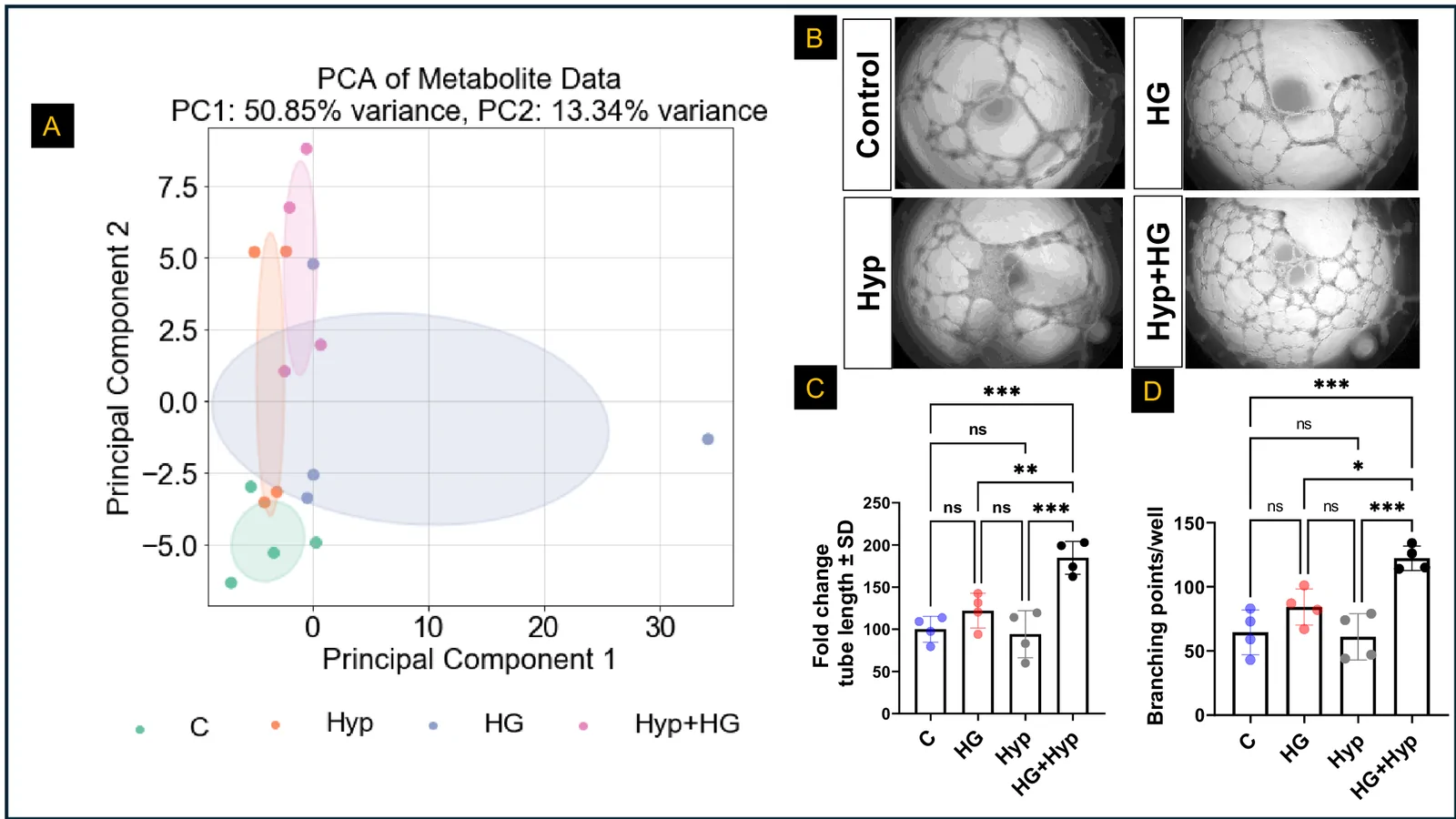
Heterogeneous Warburg-like metabolic subtypes and angiogenic responses in HRECs under HG, Hyp, and combined HG-Hyp conditions. (**A**) PCA of global metabolomic profiles in HRECs treated with normal glucose supplemented with 25 mM mannitol as an osmotic control (**C**), HG (25 mM), Hyp (2% O_2_), or combined HG-Hyp conditions, as described in the Materials and Methods. (**B**) Representative tube formation images across all conditions, demonstrating enhanced angiogenesis under combined HG-Hyp treatment relative to control, HG, or Hyp alone. Quantification of tube length (**C**) and branching points (**D**) confirmed increased angiogenic responses in the combined HG-Hyp condition. Data are presented as mean ± SD; *P < 0.05, **P < 0.01, ***P < 0.001; n = 4 per group.

**Figure 2.**
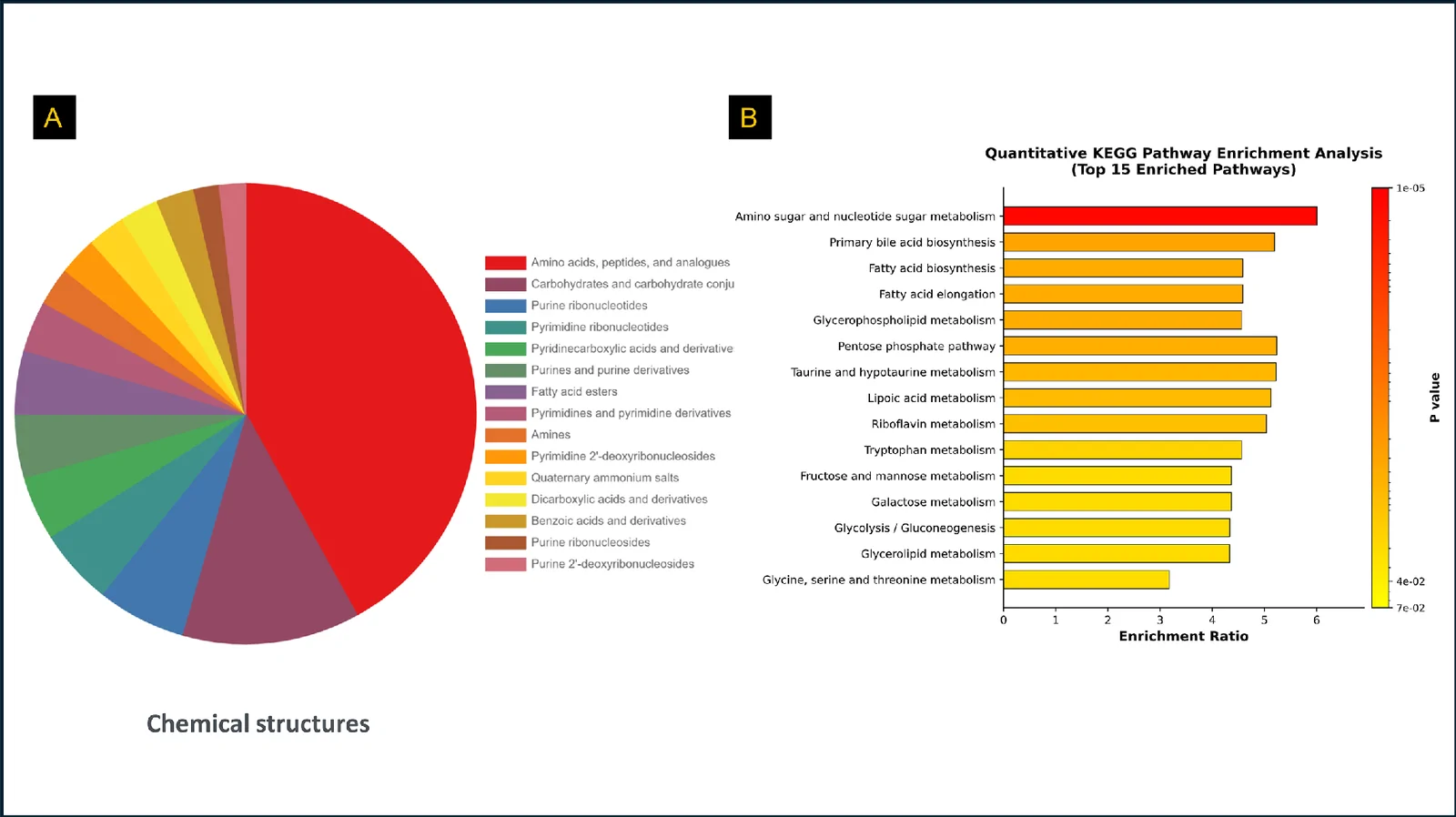
Metabolomic profiling of HRECs under combined HG-Hyp conditions. (**A**) Distribution of metabolites across different chemical classes. (**B**) Quantitative KEGG pathway enrichment analysis showing the top 15 enriched metabolic pathways ranked by enrichment ratio. Bar color represents pathway significance (*P* value).

We previously demonstrated that HG, Hyp, and their combination induce metabolic features characteristic of the Warburg-like metabolic reprogramming in HRECs, as evidenced by elevated lactate production and increased glucose uptake.^17^ Despite these shared metabolic hallmarks, PCA of LC-MS/MS-based global metabolomics, covering carbohydrates, amino acids, nucleotides, and lipids, revealed partial separation into condition-specific metabolic signatures (Fig. 1A). These findings suggest the presence of heterogeneous and partially overlapping Warburg-like metabolic subtypes that may be associated with different biological outcomes. Further analysis revealed that the combined HG-Hyp condition notably enhanced angiogenesis (Fig. 1B), evidenced by significant increases in endothelial tube length and branching points (Figs. 1C, 1D, respectively) compared to HG or Hyp alone, consistent with our recent report.^18^ To further investigate the metabolic pathways underlying the angiogenic response induced by the combined effects of HG and Hyp, LC-MS/MS-based metabolomic data were analyzed using MetaboAnalyst 6.0. Chemical structure classification of the detected metabolites revealed that amino acids, peptides, and analogues constituted the largest metabolite class, followed by carbohydrates and carbohydrate conjugates, with purine and pyrimidine ribonucleotides also representing major components of the metabolomic profile (Fig. 2A). These metabolite classes reflect the increased biosynthetic and energetic demands associated with endothelial proliferation by providing substrates for protein synthesis, carbohydrate metabolism, and nucleotide biosynthesis required to support angiogenesis.^16^ To complement this analysis, quantitative KEGG pathway enrichment analysis was performed to identify coordinated metabolic alterations associated with combined HG and Hyp treatment. Among the top 15 enriched pathways (Fig. 2B), several are directly relevant to endothelial angiogenesis, including amino sugar and nucleotide sugar metabolism, fatty acid biosynthesis, fatty acid elongation, glycerophospholipid metabolism, the pentose phosphate pathway, and glycolysis/gluconeogenesis. Amino sugar and nucleotide sugar metabolism supports the synthesis of glycosaminoglycans, which are essential for extracellular matrix remodeling and endothelial cell migration.^31^ A coordinated lipid metabolic program comprising fatty acid biosynthesis, fatty acid elongation, and glycerophospholipid metabolism was also significantly enriched, reflecting the increased demand for lipid precursors required for membrane biogenesis during endothelial proliferation, migration, and tube formation.^32^^,^^33^ The pentose phosphate pathway was significantly enriched, consistent with its well-established dual role in supplying ribose-5-phosphate for de novo nucleotide biosynthesis and generating NADPH to maintain cellular reductive capacity.^34^ Glycolysis/gluconeogenesis was likewise enriched, further supporting the Warburg-like hyperglycolytic phenotype previously demonstrated in HRECs exposed to the same experimental conditions.^17^ Together, these findings indicate the presence of specific metabolic adaptations that enhance angiogenesis in HRECs experiencing Warburg-like metabolic reprogramming induced by the combined HG and Hyp conditions.

### Machine Learning Models for Identifying the Warburg-Like Metabolic Subtypes

Building on our prior work showing that the Warburg-like metabolic reprogramming in HRECs comprises different subtypes driven by PDR-associated risk factors,^17^^,^^18^ we next assessed whether these subtypes could be more accurately distinguished using supervised machine-learning approaches. PCA of the metabolomic profiles revealed partial separation among conditions (Fig. 1A), consistent with the limitations of this unsupervised method in capturing complex, multi-feature relationships in high-dimensional data. Therefore we applied a panel of supervised classifiers (decision tree, logistic regression, naïve Bayes, random forest, KNN, neural network, gradient boosting, AdaBoost, and SVM) to leverage multivariate structure and identify discriminative metabolic patterns not evident in PCA space.

Model performance was first evaluated using 10-fold cross-validation to estimate classifier generalizability (Table 2). Among the evaluated models, AdaBoost showed high overall performance (AUC = 0.793, CA = 0.719, F1 = 0.715, MCC = 0.632, log-loss = 1.198), reflecting balanced classification and minimal misclassification. Gradient boosting achieved similarly robust performance (AUC = 0.852, CA = 0.625, F1 = 0.626, MCC = 0.501, log-loss = 1.002). Naïve Bayes demonstrated a high AUC (0.869) and good predictive metrics (CA = 0.719, F1 = 0.724, MCC = 0.633, log-loss = 0.888), suggesting good separability of subtypes.

**Table 2.** Performance of Supervised Machine-Learning Models Using 10-Fold Cross-Validation

Model	AUC	CA	F1	Precision	Recall	MCC	Specificity	LogLoss
AdaBoost	0.793	0.719	0.715	0.744	0.719	0.632	0.906	1.198
Gradient boosting	0.852	0.625	0.626	0.629	0.625	0.501	0.875	1.002
Logistic regression	0.421	0.250	0.100	0.062	0.250	0.000	0.750	1.887
KNN	0.723	0.375	0.362	0.370	0.375	0.170	0.792	2.064
Random forest	0.785	0.594	0.586	0.605	0.594	0.466	0.865	1.090
SVM	0.453	0.375	0.334	0.315	0.375	0.177	0.792	1.449
Naïve Bayes	0.869	0.719	0.724	0.762	0.719	0.633	0.906	0.888
Neural network	0.793	0.562	0.558	0.565	0.562	0.419	0.854	1.106
Decision tree	0.607	0.219	0.167	0.145	0.219	−0.045	0.740	9.790

Random forest showed moderate performance (AUC = 0.785, CA = 0.594, F1 = 0.586, MCC = 0.466, log-loss = 1.090). KNN performed less effectively (AUC = 0.723, CA = 0.375, F1 = 0.362, MCC = 0.170, log-loss = 2.064), indicating challenges in capturing high-dimensional feature interactions. The neural network achieved a high AUC (0.793) but moderate CA, F1, and MCC (0.562, 0.558, and 0.419, respectively).

Decision tree performed poorly across metrics (AUC = 0.607, CA = 0.219, F1 = 0.167, MCC = −0.045, log-loss = 9.790), with its markedly elevated log-loss reflecting high-confidence misclassifications rather than merely inconsistent predictions. Logistic regression (AUC = 0.421, CA = 0.250, F1 = 0.100, MCC = 0.000, log-loss = 1.887) exhibited limited predictive capability, likely reflecting its inability to capture non-linear relationships in the metabolomic data. SVM (AUC = 0.453, CA = 0.375, F1 = 0.334, MCC = 0.177, log-loss = 1.449) likewise showed limited predictive capability, which may reflect the small sample size relative to feature dimensionality rather than kernel limitations, given its use of a non-linear radial basis function kernel.

To further evaluate the model performance and address concerns related to data partitioning in a limited sample size, all classifiers were additionally assessed using LOOCV (Table 3). The observed trends under LOOCV were largely consistent with those obtained using 10-fold cross-validation, indicating stable model behavior. Under LOOCV, AdaBoost remained one of the top-performing models (AUC = 0.793, CA = 0.719, F1 = 0.712, MCC = 0.636), closely followed by gradient boosting (AUC = 0.840, CA = 0.625, F1 = 0.626, MCC = 0.501), naïve Bayes (AUC = 0.844, CA = 0.656, F1 = 0.668, MCC = 0.551), random forest (AUC = 0.770, CA = 0.625, F1 = 0.601, MCC = 0.521), and the neural network (AUC = 0.783, CA = 0.594, F1 = 0.586, MCC = 0.464). KNN showed moderate performance (AUC = 0.736, CA = 0.469, F1 = 0.459, MCC = 0.298), whereas SVM exhibited limited discriminative ability (AUC = 0.470, CA = 0.375, F1 = 0.375, MCC = 0.172). Logistic regression (AUC = 0.000, CA = 0.250, F1 = 0.100, MCC = 0.000) and decision tree (AUC = 0.596, CA = 0.250, F1 = 0.184, MCC = 0.000) again demonstrated poor classification performance, consistent with 10-fold cross-validation. Taken together, these LOOCV results indicate that boosting-based ensemble models, particularly AdaBoost and gradient boosting, most effectively capture the complex multivariate metabolic patterns underlying the Warburg-like metabolic subtypes, whereas naïve Bayes also demonstrates strong discriminatory performance. The concordance between LOOCV and 10-fold cross-validation further indicates that model behavior is stable and not dependent on a single data-splitting strategy.

**Table 3.** Performance of Supervised Machine-Learning Models Using LOOCV

Model	AUC	CA	F1	Precision	Recall	MCC
AdaBoost	0.793	0.719	0.712	0.748	0.719	0.636
Gradient boosting	0.840	0.625	0.626	0.629	0.625	0.501
KNN	0.736	0.469	0.459	0.483	0.469	0.298
Random forest	0.770	0.625	0.601	0.713	0.625	0.521
SVM	0.470	0.375	0.375	0.404	0.375	0.172
Naïve Bayes	0.844	0.656	0.668	0.720	0.656	0.551
Neural network	0.783	0.594	0.586	0.599	0.594	0.464
Decision tree	0.596	0.250	0.184	0.175	0.250	0.000
Logistic regression	0.000	0.250	0.100	0.062	0.250	0.000

**Figure 3.**
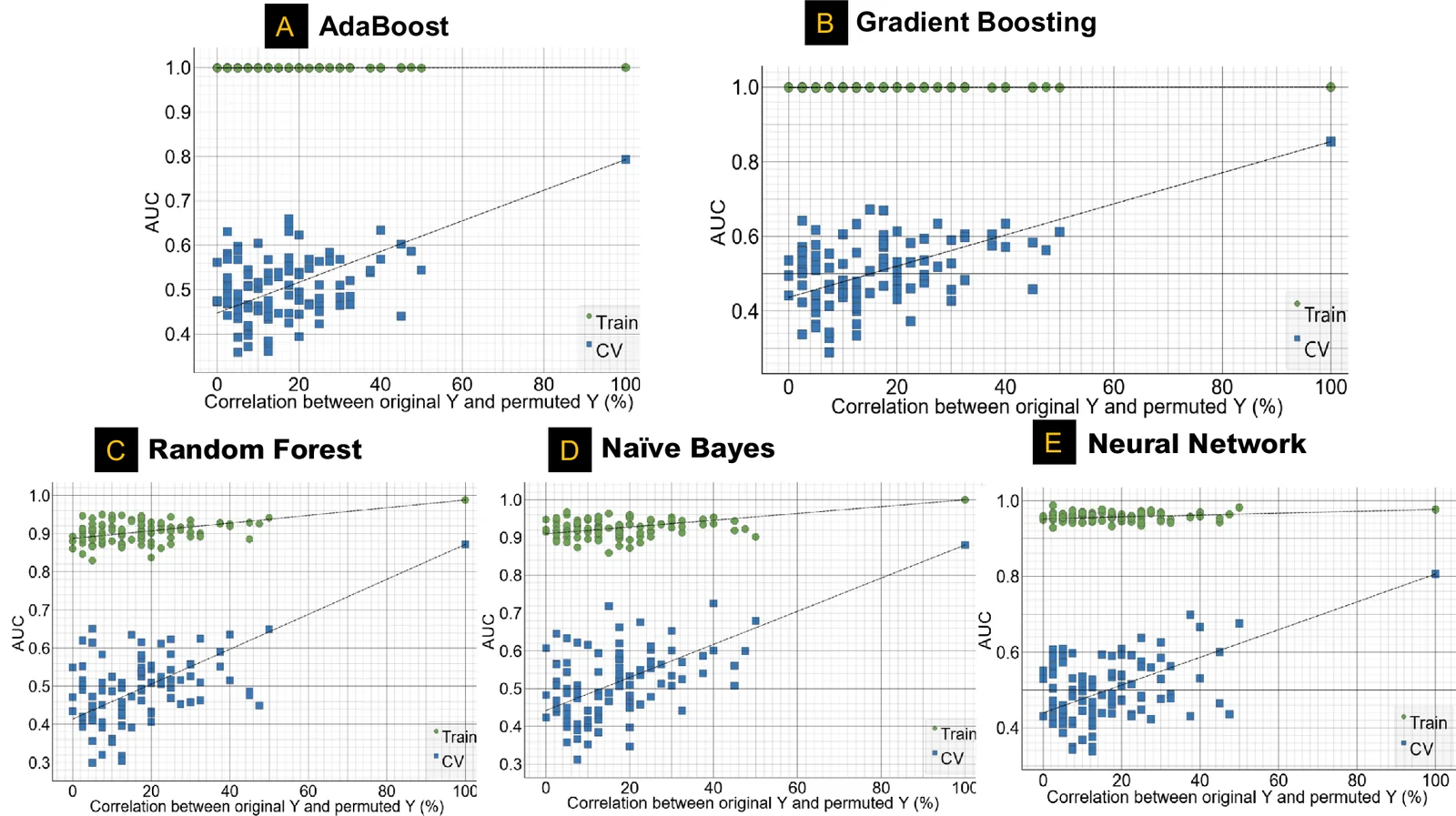
Permutation analysis validating classifiers' ability to distinguish the Warburg-like metabolic subtypes. Permutation analysis showing cross-validated (CV) AUC (*blue squares*) and training AUC (*green circles*) plotted against the correlation between original and permuted class labels (0-100%), for AdaBoost (**A**), gradient boosting (**B**), random forest (**C**), naïve Bayes (**D**), and neural network (**E**) under 100 random label shuffles, demonstrating a substantial reduction in CV AUC compared with true-label conditions and confirming that observed discriminative performance exceeds random expectation.

To further confirm that the observed classification performance of the top-performing models exceeded random expectation, permutation testing was applied to AdaBoost, gradient boosting, random forest, naïve Bayes, and the neural network. All five models showed a substantial numerical reduction in cross-validated performance under permutation compared with true-label conditions (Fig. 3; Table 4), demonstrating loss of true discriminative structure. For AdaBoost, the cross-validated AUC decreased from 0.793 with true labels to 0.448 after permutation, representing a large drop in discriminative ability and indicating that its predictive performance is highly unlikely to arise by chance. Similar pronounced losses in performance were observed for gradient boosting (AUC: 0.854 to 0.437), Random Forest (AUC: 0.872 to 0.414), Naïve Bayes (AUC: 0.880 to 0.442), and the Neural Network (AUC: 0.806 to 0.440). These permutation results, together with the concordant performance observed across 10-fold cross-validation and LOOCV, demonstrate that classifier discrimination exceeds random expectation and collapses under label randomization, indicating that the observed metabolic signatures distinguishing the Warburg-like metabolic subtypes are reproducible within this dataset and warrant further validation for biological relevance.

**Table 4.** AUC Under Permutation (*n* = 100 Permutations)

Model	Train AUC (Corr = 0%)	Train AUC (Corr = 100%)	CV AUC (Corr = 0%)	CV AUC (Corr = 100%)
AdaBoost	0.9989	1.0000	0.4475	0.7930
Gradient boosting	0.9988	1.0000	0.4366	0.8542
Random forest	0.8873	0.9883	0.4139	0.8717
Naïve Bayes	0.9099	1.0000	0.4419	0.8802
Neural network	0.9520	0.9766	0.4398	0.8060

### Machine Learning Models for Identifying the Warburg-Like Metabolic Subtypes Under Domain Shift

#### Analysis of AUC

**Figure 4.**
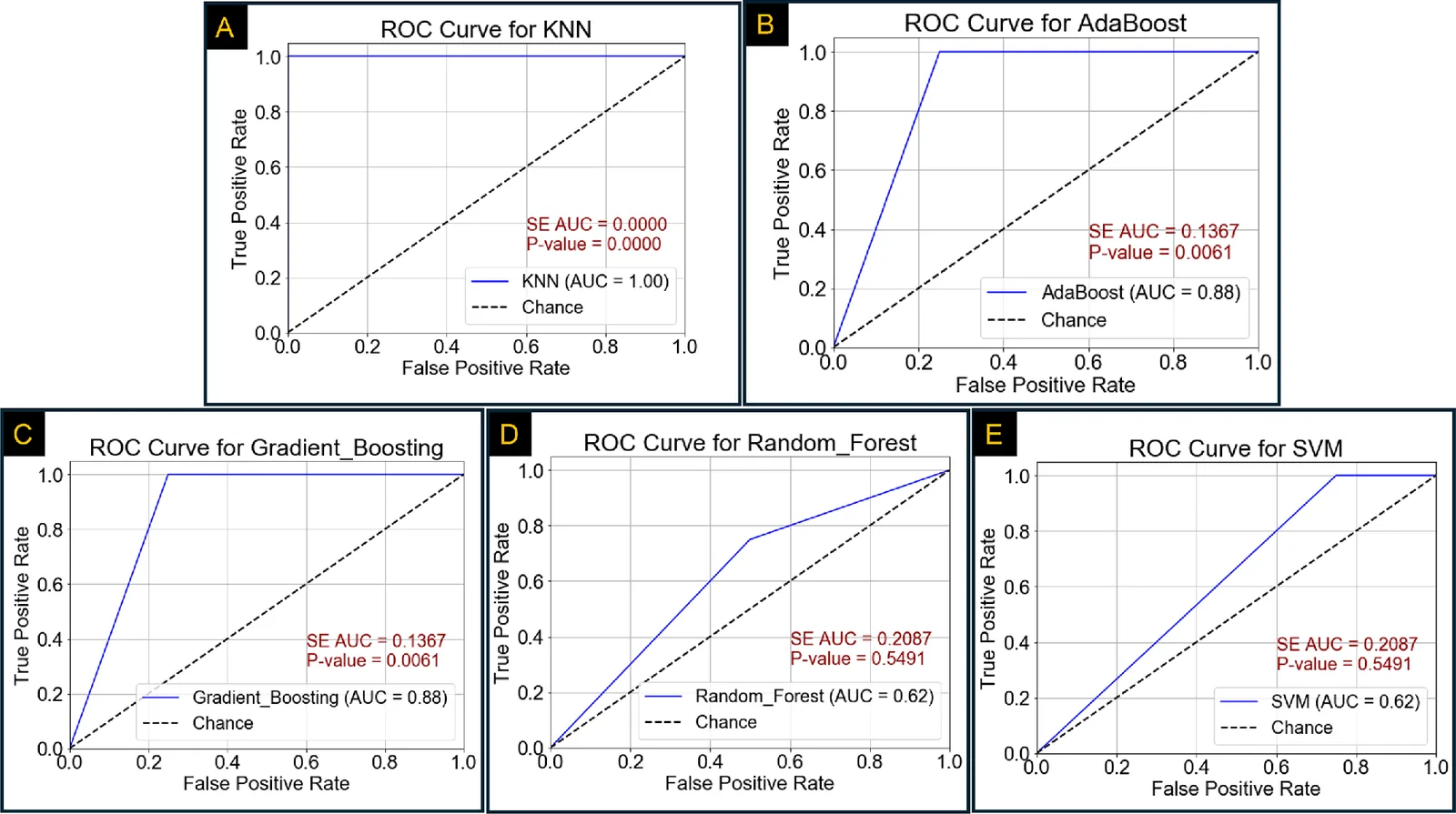
Receiver operating characteristic curves of top-performing machine learning models for distinguishing the Warburg-like metabolic subtypes under domain-shift conditions (CoCl_2_-induced pseudo-hypoxia). (**A**) KNN. (**B**) AdaBoost. (**C**) Gradient boosting. (**D**) Random forest. (**E**) SVM.

**Figure 5.**
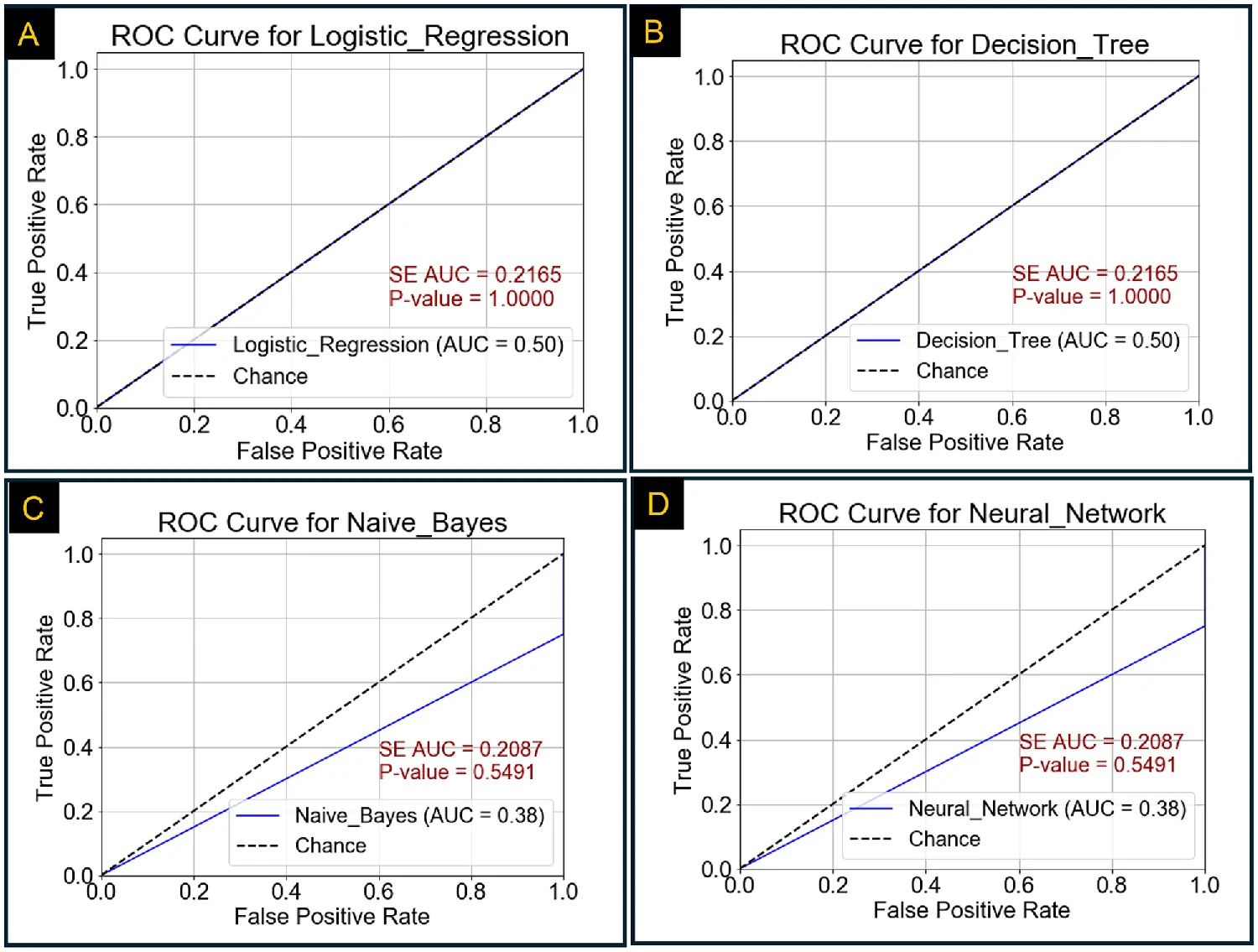
Receiver operating characteristic curves of lower-performing classifiers for distinguishing the Warburg-like metabolic subtypes under domain-shift conditions. (**A**) Logistic regression. (**B**) Decision tree. (**C**) Naïve Bayes. (**D**) Neural network.

These analyses were conducted as an exploratory stress test of classifier stability under biologically meaningful distributional shift, assessing whether models trained on physiological hypoxia (2% O_2_) retain discriminative performance between the Warburg-like metabolic subtypes when tested on an independent dataset generated using a mechanistically distinct hypoxia-inducing agent (CoCl_2_). As shown in Figures 4 and 5, classifier performance varied considerably under these conditions. KNN achieved apparent perfect discrimination (AUC = 1.00; Fig. 4A); however, this apparent optimal performance is most consistent with overfitting, given its substantially lower performance across internal validation strategies (Tables 2, 3).

**Figure 6.**
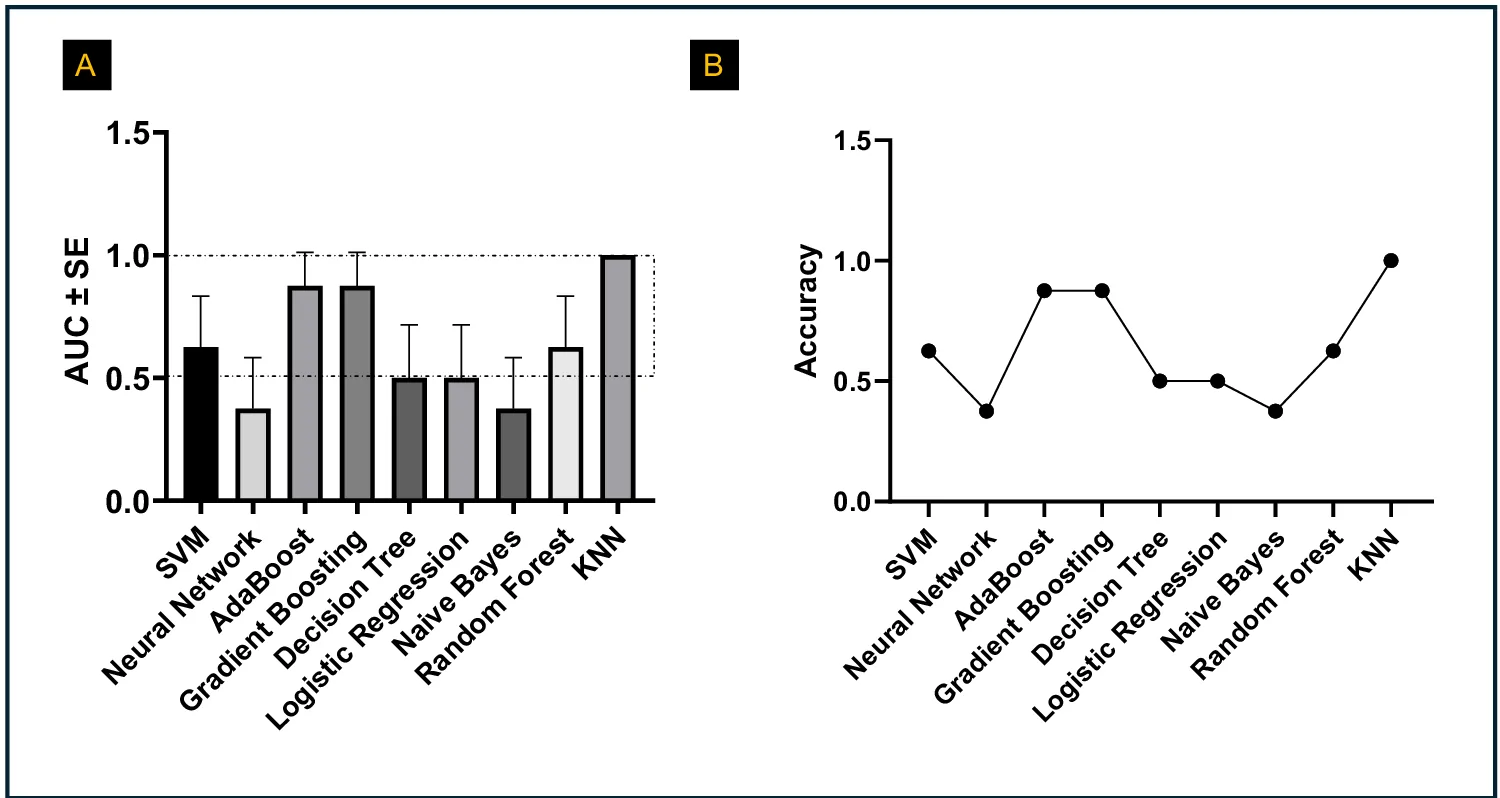
Overall performance metrics of machine learning models for distinguishing the Warburg-like metabolic subtypes under domain shift. (**A**) AUC ± SE values. (**B**) CA of each model.

In contrast, boosting-based ensemble learning methods demonstrated statistically reliable performance under domain shift. Both AdaBoost and gradient boosting achieved an AUC of 0.88 (Figs. 4B, 4C), with significance testing confirming performance above random expectation (*P* = 0.0061). This indicates that these two models retained discriminative capacity across mechanistically distinct hypoxia-inducing conditions. Random forest and SVM showed only modest discrimination (AUC = 0.62; Figs. 4D, 4E), with performance not significantly above chance (*P* = 0.5491). Logistic regression and decision tree offered no discriminative capability (AUC = 0.500; *P* = 1.0; Figs. 5A, 5B). Naïve Bayes and the neural network performed poorly (AUC = 0.38; Figs. 5C, 5D; *P* = 0.5491), indicating limited sensitivity to the metabolic reprogramming induced by CoCl_2_-mediated hypoxia. Collectively, these findings show that while KNN may display inflated performance due to overfitting, AdaBoost and gradient boosting maintain predictive performance under domain shift. AUC ± SE values are summarized in Figure 6A.

#### Analysis of Accuracy

The overall CA is presented in Figure 6B. Consistent with its AUC, KNN reached apparent perfect accuracy (CA = 100%). AdaBoost and gradient boosting achieved CA = 87.5%, representing the strongest generalizable performance among non-overfit models. SVM and random forest each demonstrated moderate accuracy (62.5%). Logistic regression and decision tree performed at the chance level (50%). Neural network and naïve Bayes, both at 37.5% accuracy, showed the poorest ability to classify the Warburg-like metabolic subtypes under domain shift.

#### Analysis of Confusion Matrices

**Figure 7.**
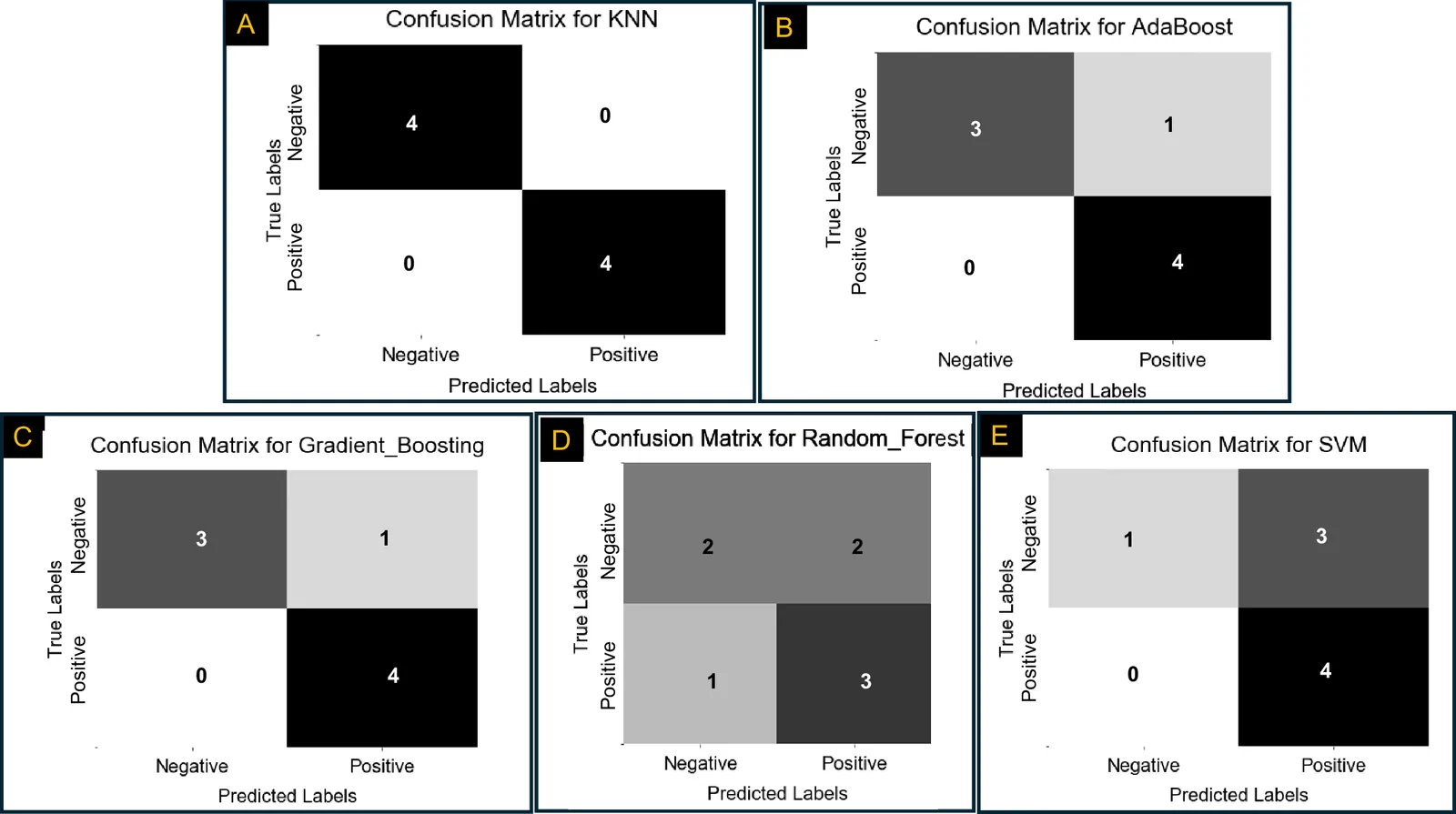
Confusion matrices for models with AUC > 0.5 under domain-shift conditions for distinguishing the Warburg-like metabolic subtypes (HG-Hyp as positive class, Hyp-alone as negative class). (**A**) KNN. (**B**) AdaBoost. (**C**) Gradient boosting. (**D**) Random forest. (**E**) SVM.

To further evaluate model behavior under domain shift, confusion matrices were generated using the HG-Hyp subtype as the positive class and the Hyp-alone subtype as the negative class. Among high-performing models (AUC > 0.5), AdaBoost demonstrated comparatively strong and balanced discrimination, correctly classifying most samples of both subtypes, although some misclassification remained (Fig. 7B). Gradient boosting showed a highly similar and comparably balanced pattern (Fig. 7C). Random forest displayed moderate discrimination, with misclassification present in both classes (Fig. 7D), whereas SVM showed moderate discrimination driven primarily by misclassification of the negative class, despite correctly identifying all true-positive samples (Fig. 7E). KNN achieved perfect separation (Fig. 7A), but in light of its poor generalizability across internal validation schemes, this pattern likely reflects overfitting to local sample structure rather than genuine biological discrimination.

**Figure 8.**
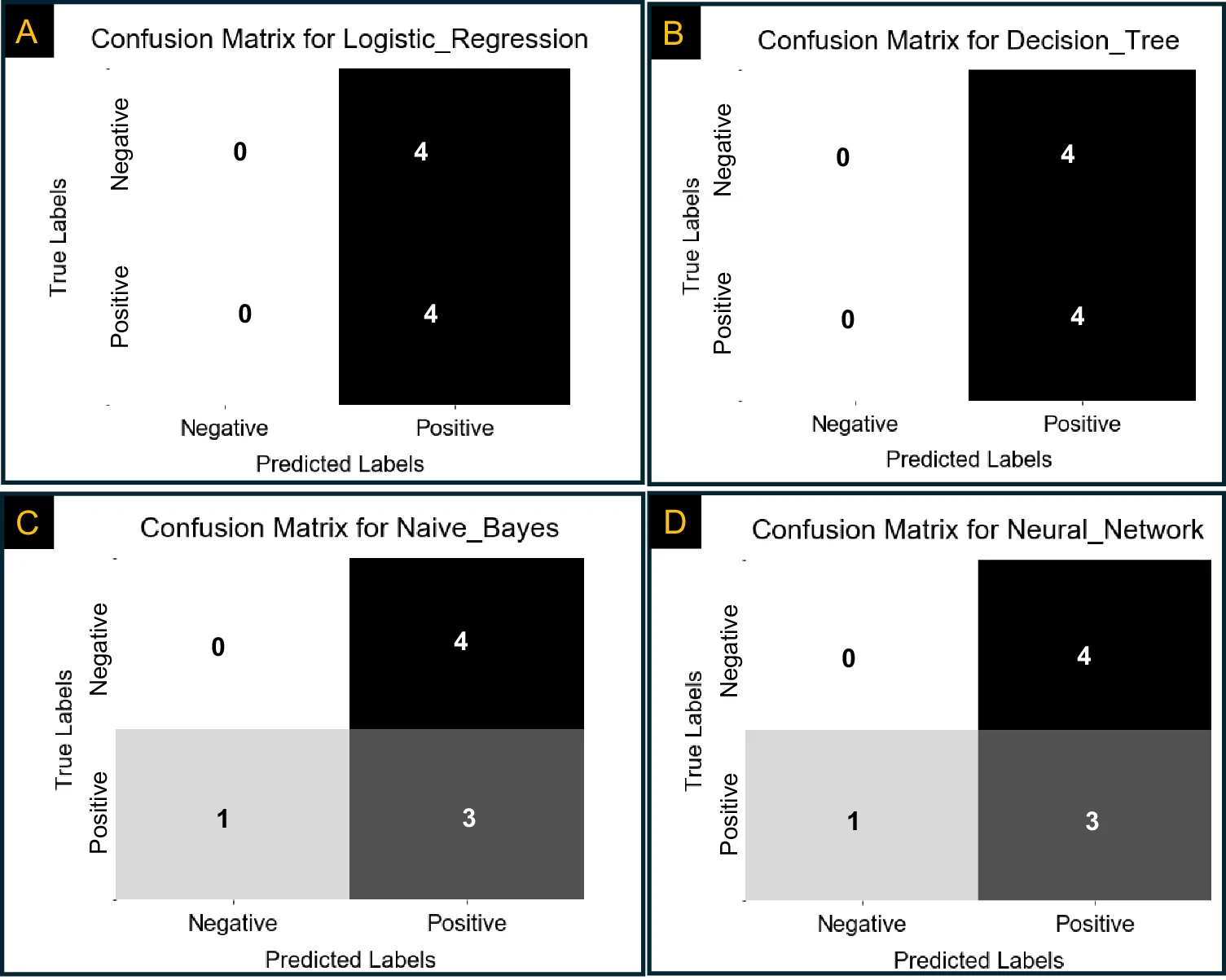
Confusion matrices for models with AUC ≤ 0.5 under domain-shift conditions for distinguishing the Warburg-like metabolic subtypes. (**A**) Logistic regression. (**B**) Decision tree. (**C**) Naïve Bayes. (**D**) Neural network.

Among the weak-performing classifiers, logistic regression and decision tree collapsed into single-class prediction, failing to distinguish the two Warburg-like metabolic subtypes (Figs. 8A, 8B). Naïve Bayes and the neural network showed poor discrimination, driven predominantly by complete misclassification of the negative class despite correctly identifying most true-positive samples (Figs. 8C, 8D), consistent with their poor AUC performance. Overall, the confusion-matrix patterns support the use of boosting-based ensembles, particularly AdaBoost and gradient boosting, in detecting the Warburg-like metabolic subtypes under domain-shift conditions.

#### Analysis of Sensitivity

**Figure 9.**
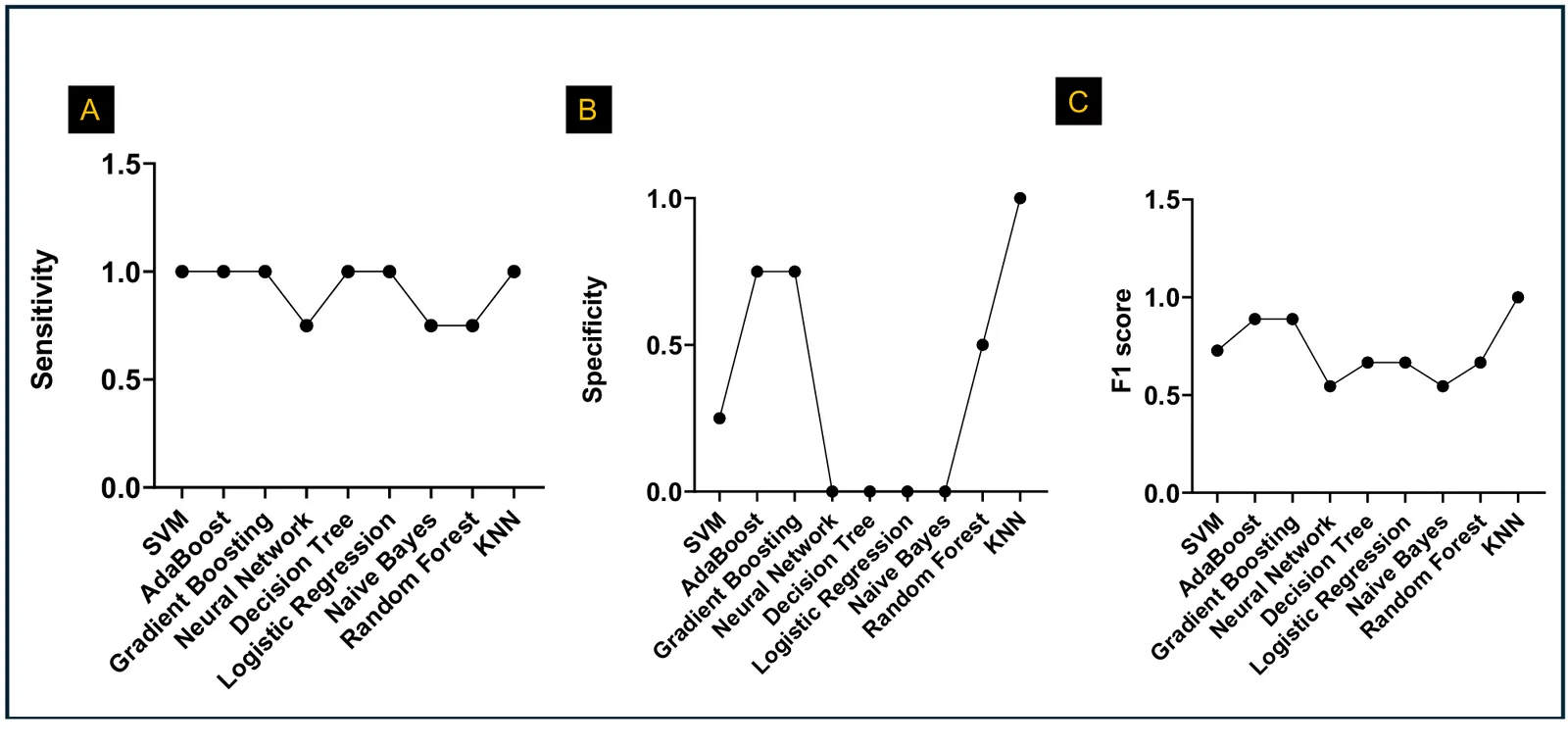
Sensitivity, specificity, and F1 score of machine learning models under domain-shift conditions for distinguishing the Warburg-like metabolic subtypes. (**A**) Sensitivity (true positive rate), showing that most models detect the HG-Hyp subtype. (**B**) Specificity (true negative rate), highlighting deficits in correctly classifying Hyp-alone samples for several models. (**C**) F1 scores, reflecting the harmonic mean of precision and recall, highlight AdaBoost and gradient boosting as the most balanced classifiers under domain shift.

Sensitivity, or the true positive rate, measures a model's ability to correctly identify positive cases (the Warburg-like metabolic subtype induced by HG-Hyp). As shown in Figure 9A, most classifiers, including SVM, AdaBoost, gradient boosting, decision tree, logistic regression, and KNN, achieved perfect sensitivity (1.00), correctly identifying all true-positive samples. Neural network, naïve Bayes, and random forest each exhibited moderately high sensitivity (0.75), indicating partial but incomplete detection of the HG-Hyp subtype. Overall, these results indicate that failure to detect positive cases was not a major source of performance limitation for most models, suggesting that differences in overall performance were driven primarily by other factors, such as reduced specificity or misclassification of negative samples.

#### Analysis of Specificity

Specificity or true negative rate reflects the ability to correctly classify negative cases (not the Warburg-like metabolic subtype caused by HG-Hyp). As shown in Figure 9B, KNN achieved perfect specificity (1.00). AdaBoost and gradient boosting followed with a specificity of 0.75, and random forest achieved 0.50. In contrast, SVM showed low specificity (0.25), and neural network, decision tree, logistic regression, and naïve Bayes each demonstrated zero specificity, misclassifying all negative samples. These findings show that although many models identify HG-Hyp, several exhibit significant specificity deficits.

#### Analysis of F1 Score

The F1 score, which balances precision and recall, provides an integrated assessment of overall classifier performance. As shown in Figure 9C, KNN achieved the highest F1 score (1.00), reflecting a perfect balance between true-positive and true-negative classification. However, given KNN's instability across internal validation schemes (Tables 2, 3), this apparent optimal performance is most consistent with overfitting to the small CoCl_2_ test set rather than true generalizability. AdaBoost and gradient boosting showed reliable performance, each with an F1 score of 0.89, supported by perfect sensitivity and high specificity. Logistic regression, decision tree, and SVM yielded intermediate-to-high F1 scores (0.67–0.73) driven primarily by high sensitivity coupled with low specificity, rather than a balanced trade-off between subtype identification and false-positive control. Random forest showed a comparable F1 score (0.67) despite more moderate sensitivity and specificity values (Figs. 9A, 9B), indicating a more evenly distributed error pattern across both classes. Neural network and naïve Bayes exhibited lower F1 values (0.55), consistent with their limited specificity. Together, these results indicate that while several models can detect positive cases, the boosting-based algorithms demonstrated relatively better performance in balancing true-positive detection and false-positive control under domain-shift conditions.

## Discussion

This study provides evidence supporting the heterogeneity of the Warburg-like metabolic reprogramming in HRECs, suggesting the presence of metabolically distinct states associated with PDR-related risk factors, including HG, Hyp, and their combination. Although prior work demonstrated that these conditions induce shared Warburg-like metabolic features,^17^ our current analyses indicate that the combined HG-Hyp condition is associated with a distinct metabolic profile accompanied by increased angiogenic activity. By integrating metabolomic profiling with supervised machine-learning analyses, these metabolic states were distinguishable within the current experimental framework. This interpretation is supported by several observations: (1) PCA of LC-MS/MS metabolomics revealed partial separation of metabolic signatures across conditions, indicating heterogeneity among the Warburg-like metabolic profiles; (2) The combined HG-Hyp condition significantly enhanced endothelial tube formation and branching relative to either stressor alone; (3) Pathway enrichment analysis identified metabolic pathways, including amino sugar, nucleotide sugar, fatty acid, glycerophospholipid, and pentose phosphate metabolism, that are known to support angiogenic processes; and (4) Supervised machine-learning models identified multivariate metabolic patterns that discriminated these conditions, with boosting-based ensemble methods (AdaBoost and gradient boosting) demonstrating the most balanced and reproducible performance across different validation schemes. To our knowledge, this study represents one of the first applications of machine-learning approaches to explore the Warburg-like metabolic heterogeneity in retinal endothelial angiogenesis under PDR-related risk factors.

Across multiple validation strategies, boosting-based ensemble classifiers demonstrated the most consistent performance in distinguishing the Warburg-like metabolic subtypes. Under 10-fold cross-validation, AdaBoost achieved balanced classification metrics (AUC = 0.793, CA = 0.719, F1 = 0.715, MCC = 0.632), whereas gradient boosting showed comparable discriminative ability with slightly reduced classification accuracy but higher AUC (0.852). Importantly, these performance trends were preserved under LOOCV, with minimal deviation in AUC, CA, F1, and MCC values. Random forest also demonstrated moderate and stable performance across 10-fold cross-validation and LOOCV, but did not exceed the discriminative performance observed for AdaBoost and gradient boosting. This concordance between 10-fold cross-validation and LOOCV suggests that boosting-based ensemble models capture reproducible multivariate metabolic patterns rather than exploiting sample-specific noise. These findings are consistent with prior studies indicating that AdaBoost and gradient boosting are well suited for modeling complex, non-linear relationships in high-dimensional datasets, including metabolomic profiles.^35^ AdaBoost, in particular, uses an iterative reweighting strategy that emphasizes misclassified samples,^24^ which may contribute to its comparatively strong and stable performance observed in this study.

Naïve Bayes also demonstrated relatively strong internal performance, achieving high AUC values and favorable classification metrics under both 10-fold cross-validation and LOOCV. However, its performance deteriorated substantially under domain-shift conditions, indicating limited robustness when applied to biologically distinct hypoxia models. This discrepancy likely reflects the conditional independence assumptions inherent to naïve Bayes, which may be satisfied within a single experimental distribution but can break down when metabolite dependencies change across biological contexts.^36^

In contrast, simpler classifiers such as logistic regression and decision tree showed limited discriminative capability across validation schemes, performing at or near chance levels in terms of classification accuracy and correlation-based metrics. Logistic regression demonstrated lower discriminative ability (AUC = 0.421 under 10-fold cross-validation and complete collapse under LOOCV), consistent with its assumption of linear relationships among features,^37^^–^^39^ which is likely insufficient to capture the complex, non-linear interactions underlying the Warburg-like metabolic subtypes. Decision trees also exhibited poor performance, characterized by low accuracy and negative or zero MCC values, consistent with their known susceptibility to overfitting in small, high-dimensional datasets.^40^^,^^41^

SVM and neural network classifiers exhibited variable performance across validation schemes. Neural networks achieved reasonable internal discrimination under 10-fold cross-validation and LOOCV; however, their marked loss of performance under domain-shift conditions suggests overfitting to the training distribution, likely exacerbated by limited sample size and the high dimensionality of the metabolomic feature space.^42^^,^^43^ In contrast, SVM demonstrated consistently weak and unstable performance across internal 10-fold cross-validation, LOOCV, and domain-shift analyses, indicating limited capacity to separate the Warburg-like metabolic subtypes within this metabolomic dataset. Although SVMs are generally well-suited for high-dimensional classification tasks, their effectiveness depends on clear class boundaries and appropriate kernel specification; the apparent feature overlap in the present data likely constrained their discriminative ability.^27^

Evaluation under domain-shift conditions further highlighted differences in classifier stability under biologically meaningful distributional shift. It is important to note that physiological hypoxia (2% O_2_) and CoCl_2_-induced HIF stabilization represent mechanistically distinct perturbations, the former directly limiting oxygen availability, the latter inhibiting prolyl hydroxylases independently of oxygen tension, and their precise metabolic differences are not fully characterized. This analysis therefore combines two sources of uncertainty: biological non-equivalence between the two hypoxia models and the inherent challenge of extrapolating nonlinear classifiers across distributional shifts. Accordingly, it is best interpreted as a stress test of classifier stability rather than as biological validation or evidence of metabolic equivalence between hypoxia models. Within this framework, AdaBoost and gradient boosting demonstrated the strongest retained performance (AUC = 0.88, *P* = 0.0061), consistent with a greater capacity to capture metabolic features that are shared across both hypoxia-inducing conditions, likely reflecting the convergent HIF-1α-dependent glycolytic programs activated by both 2% O_2_ and CoCl_2_, rather than overfitting to the specific distributional characteristics of either dataset. In contrast, most other models performed at or near chance levels under domain shift. Although KNN exhibited perfect classification, this result occurred alongside weaker performance in both 10-fold cross-validation and LOOCV, suggesting instance memorization rather than true generalizability.

It is also worth noting that several classifiers that showed limited performance in the present study, including KNN, SVM, and logistic regression, have been reported to achieve high diagnostic performance (AUCs ≥ 0.82) in retinal image classification tasks.^44^^–^^47^ This difference in performance likely reflects fundamental differences between retinal imaging and metabolomic datasets. Unlike retinal images, which capture disease-related information in structured spatial patterns, metabolomic data are characterized by highly interdependent biochemical features and complex nonlinear pathway interactions, which may reduce the discriminative ability of these models to distinguish the Warburg-like metabolic subtypes in the present context.

Taken together, these findings underscore the importance of evaluating machine-learning models using complementary validation strategies rather than relying on a single performance metric. Consistent behavior across 10-fold cross-validation, LOOCV, and biologically meaningful domain-shift analyses provides stronger evidence of robustness than internal validation alone. Within this framework, boosting-based ensemble models (AdaBoost and gradient boosting) demonstrated the most consistently reliable and reproducible performance. In contrast, models such as KNN and naïve Bayes illustrate the risks of overinterpretation in small datasets, as their performance was highly context-dependent, reflecting either overfitting (KNN) or sensitivity to distributional shifts (naïve Bayes).

## Conclusions

Our findings provide preliminary support for the existence of metabolically distinct Warburg-like subtypes in HRECs under PDR-associated risk factors, with the combined effects of HG and hypoxia promoting a particularly pro-angiogenic metabolic state consistent with a nonadditive convergence of Pasteur-like and Crabtree-like glycolytic programs. By integrating untargeted metabolomics with supervised machine-learning analyses, this study establishes a framework for identifying such subtypes and generating testable hypotheses regarding their biological and clinical relevance. Future studies should validate these metabolic signatures in independent larger datasets, incorporate complementary multi-omics approaches (e.g., transcriptomics or proteomics), and extend analysis to clinical specimens, such as vitreous samples from patients with PDR, to establish disease relevance and translational potential. Collectively, these findings suggest that metabolic stratification of the Warburg-like phenotypes may provide new insight into the mechanisms driving retinal angiogenesis and inform the development of biomarkers or therapeutic targets for PDR.

### Limitations and Future Directions

Despite the encouraging classification performance observed across multiple supervised machine-learning classifiers, these results should be interpreted in light of several methodological limitations. First, the relatively modest number of biological replicates (*n* = 8 per condition) compared with the dimensionality of the metabolomic dataset limits statistical power and increases susceptibility to overfitting; accordingly, these machine-learning analyses should be regarded as exploratory. Second, although several classifiers achieved perfect sensitivity in detecting the HG-Hyp Warburg-like subtype, specificity varied substantially, with some models misclassifying all negative samples. Consequently, apparently strong performance metrics, particularly perfect sensitivity or elevated F1 scores, may partially reflect characteristics of the small dataset rather than fully generalizable predictive performance. Third, metabolite feature ranking was performed across the aggregated dataset prior to cross-validation rather than being nested within each validation fold. While this approach facilitated the identification of an informative subset of metabolites, it may introduce a modest optimistic bias in model performance estimates due to potential data leakage during feature selection. Nevertheless, the concordance observed between 10-fold cross-validation and LOOCV supports the presence of reproducible multivariate metabolic patterns within this dataset, further reinforced by permutation testing confirming performance above chance expectation. The subsequent domain-shift analysis identified AdaBoost and gradient boosting as the classifiers most likely to generalize beyond the conditions on which they were trained. Future studies using larger independent cohorts, combined with fully nested feature-selection pipelines and external validation datasets, will be essential to evaluate these models' generalizability and the biological significance of the identified metabolic signatures.

## Supplementary Material

Supplement 1
