# Supplementary material for: Machine Learning and Metabolomics to Characterize Warburg-Like Metabolic Subtypes in Human Retinal Endothelial Cells Exposed to Risk Factors Associated With Proliferative Diabetic Retinopathy
Source: Transl Vis Sci Technol. 2026 Aug 26;15(8):26. doi: 10.1167/tvst.15.8.26 (PMC13533292; doi:10.1167/tvst.15.8.26)
Supplement: Supplementary file 1 [file tvst-15-8-26_s001.pdf]

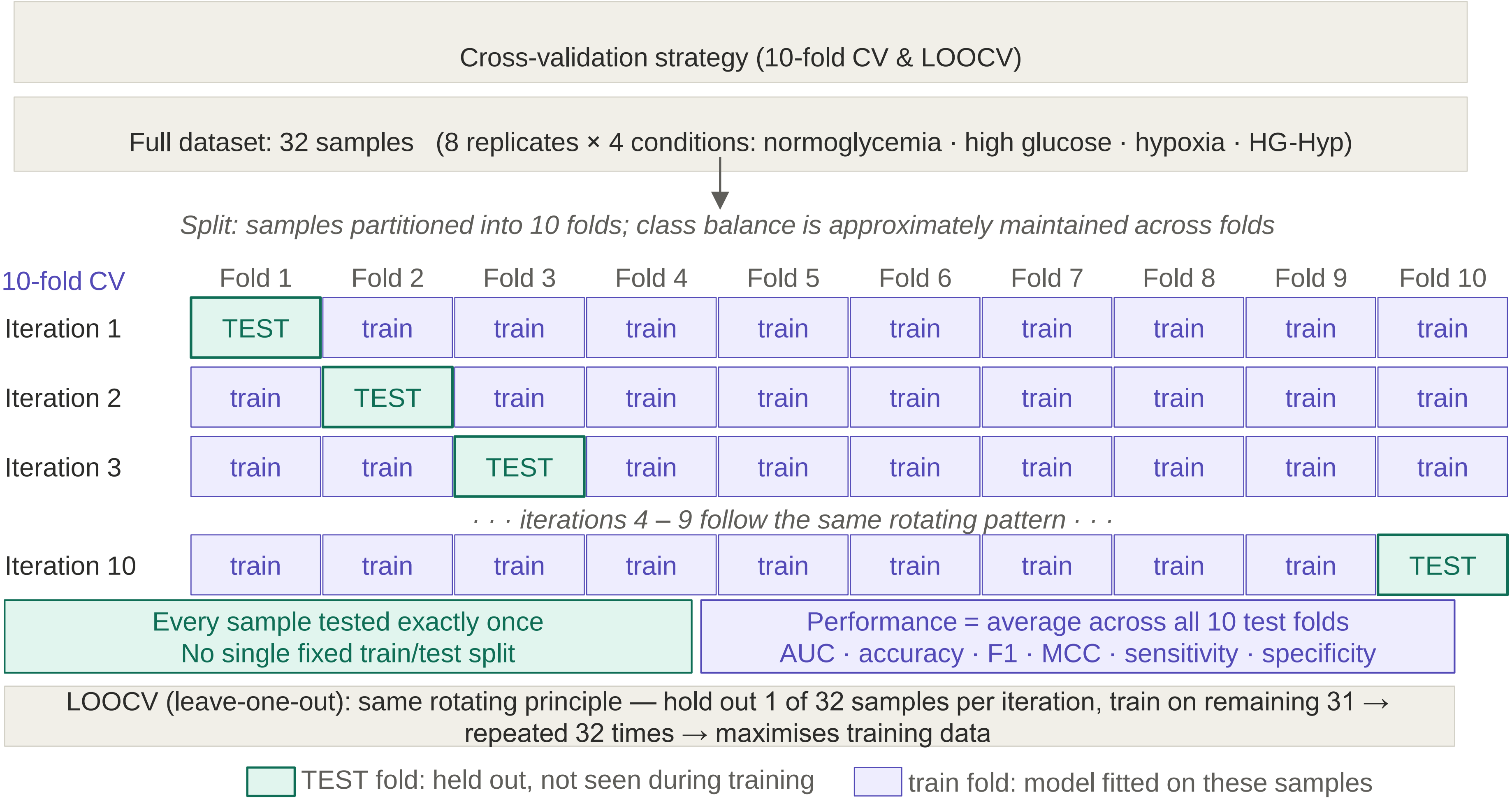


**Supplementary Figure S1.** Schematic illustration of the 10-fold cross-validation and leave-one-out cross-validation (LOOCV) strategies used for machine-learning model evaluation.
